# Our Book Table

**Published:** 1886-12

**Authors:** 


### OUR BOOK TABLE.

The usual book notices for this month are, greatly to our regret, crowded out. Among those which were prepared for the printer was a review of the initial (November) number of the new Chicago Dental Journal. Also notices of the revised and enlarged editions of Richardson's Mechanical Dentistry, Taft's Index of Dental Literature, and the Physicians' Annual Visiting List, all from P. Blakiston, Son & Co., of Philadelphia. Besides these, there are books from other publishers upon our table, which shall have attention at the earliest possible date.

